# HAPSIMU: a genetic simulation platform for population-based association studies

**DOI:** 10.1186/1471-2105-9-331

**Published:** 2008-08-05

**Authors:** Feng Zhang, Jianfeng Liu, Jie Chen, Hong-Wen Deng

**Affiliations:** 1 Institute of Molecular Genetics, School of Life Science and Technology, Xi'an Jiaotong University, Xi'an 710049, PR China; 2 Departments of Orthopedic Surgery and Basic Medical Science, School of Medicine, University of Missouri-Kansas City, Kansas City, MO 64108, USA; 3 Department of Mathematics and Statistics, University of Missouri-Kansas City, Kansas City, MO, 64110, USA; 4 Laboratory of Molecular and Statistical Genetics, College of Life Sciences, Hunan Normal University, Changsha, Hunan 410081, PR China

## Abstract

**Background:**

Population structure is an important cause leading to inconsistent results in population-based association studies (PBAS) of human diseases. Various statistical methods have been proposed to reduce the negative impact of population structure on PBAS. Due to lack of structural information in real populations, it is difficult to evaluate the impact of population structure on PBAS in real populations.

**Results:**

We developed a genetic simulation platform, HAPSIMU, based on real haplotype data from the HapMap ENCODE project. This platform can simulate heterogeneous populations with various known and controllable structures under the continuous migration model or the discrete model. Moreover, both qualitative and quantitative traits can be simulated using additive genetic model with various genetic parameters designated by users.

**Conclusion:**

HAPSIMU provides a common genetic simulation platform to evaluate the impact of population structure on PBAS, and compare the relative performance of various population structure identification and PBAS methods.

## Background

Population-based association studies (PBAS) are powerful for disease gene mapping, and are widely applied to the identification of genetic determinant of human diseases [1,2]. However, it is still an issue as to how to effectively evaluate and reduce the negative impact of population structure on PBAS [1,3].

Population structure, a common feature in real populations [4,5], is an important cause leading to inconsistent results in PBAS [1,6]. Various statistical methods have been proposed to reduce the negative impact of population structure on PBAS, [7-10]. Because of different hypotheses and algorithms, the performance of these PBAS methods may be different in different situations. Therefore, a comparison of the relative performance of various PBAS methods in heterogeneous populations may provide a practical guideline for empirical researchers to choose proper study methods which are best suitable for their respective situations, and make appropriate interpretation of their results.

Due to lack of structural information in real populations, it is difficult or impossible to accurately evaluate the impact of population structure on PBAS in real populations. Simulation, which can generate heterogeneous populations with known structures, is therefore an alternative choice for the studies aforementioned. Currently, several genetic simulation programs are available [11,12]. Most of these programs can simulate only genotype data, and not phenotype data. Furthermore, very few of these programs can generate heterogeneous populations with various known and controllable structures. Therefore, it is difficult to apply them to evaluate the impact of population structure on PBAS. To address the problems discussed above, we developed a genetic simulation platform, HAPSIMU, based on real haplotype data from the HapMap ENCODE project [see Additional file 1].

## Methods

### Genotype simulation

**Figure 1:**
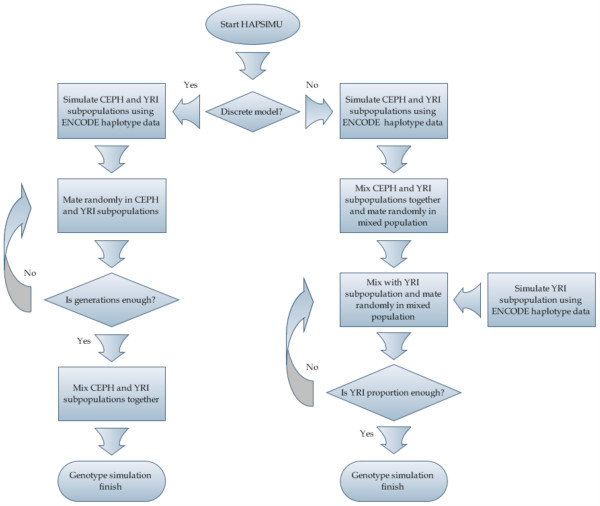
Flowchart that illustrates the simulation approach of heterogeneous populations.

The HapMap ENCODE project genotyped dense sets of SNPs across ten 500 kb regions in four populations. Phased haplotype data of Caucasian with northern and western European ancestry (CEPH) and Yoruba from Ibadan (YRI) of Africa were downloaded from HapMap ENCODE website . Within each ENCODE region, we selected the set of informative marker loci that were genotyped in both CEPH and YRI and were polymorphic in at least one population or monomorphic, but had different alleles in the two populations. There were 12,867 highly informative marker loci selected from 10 ENCODE regions. We converted the genetic map distances reported by the HapMap ENCODE project to recombination fractions between adjacent informative marker loci using the Kosambi map function [13]. Based on the phased CEPH and YRI haplotype data and derived recombination fractions for the informative marker loci, 1000 CEPH individuals and 1000 YRI individuals will be first simulated and used as CEPH and YRI founder populations. Then, heterogeneous populations composed of CEPH and YRI will be simulated under two selectable population admixture models: the continuous migration model and the discrete model [14]. As illustrated in Figure 1, under the continuous migration model, in each generation, the simulated heterogeneous population (1000 children from previous generation) will be mixed with the simulated YRI subpopulation (1000 individuals) according to users designated proportions, and then mate randomly and produce offspring in the mixed population to generate a new heterogeneous population with 1000 individuals. This simulation procedure will continue until the proportion of YRI in the simulated heterogeneous population reach the admixture proportions designated by users. Under the discrete model, the simulated CEPH (1000 individuals) and YRI (1000 individuals) subpopulations will separately, randomly mate and produce offspring for users designated generations. During this process, population size will be kept constant. Finally, the simulated CEPH and YRI subpopulations will be mixed together according to the proportions assigned by users. We assume that all markers were under Hardy-Weinberg equilibrium and randomly recombined according to the derived recombination fractions in both admixture models.

### Phenotype simulation

Additive genetic model is implemented in HAPSIMU to simulate qualitative and quantitative. For qualitative trait, the relationship among population prevalence (K), genotype relative risk (GRR) (r), frequency of causal allele (p) and penetrance (f_i_) of genotype at a causal locus in simulated heterogeneous populations can be expressed as:

f_0 _= K/(1-2p+2pr),

f_1 _= rf_0_,

f_2 _= 2rf_0_-f_0_,

where f_i _denotes the penetrance of the genotypes at the causal locus with i copy (copies) of the disease susceptible allele (i = 0, 1 or 2). For quantitative trait, the additive genetic effect of quantitative trait loci (QTL) j (a_j_) is given by:

$$a_{j}=\sqrt{\frac{V_{j}}{2p_{j}(1-p_{j})}}$$

where V_j _denotes the phenotypic variation explained by the QTL j, and p_j _denotes the frequency of the disease susceptible allele at the QTL j.

## Results

HAPSIMU can simulate heterogeneous populations with various known population structures under the continuous migration model or the discrete model. In the continuous migration model, population structure is controlled by the admixture proportion of YRI in the simulated heterogeneous populations. In the discrete model, frequency difference of disease susceptible allele(s) between the simulated CEPH and YRI subpopulations, proportions of CEPH and YRI in cases and controls (for qualitative trait) or variance explained by population stratification (for quantitative trait) can be preset by users to simulate heterogeneous populations. Additionally, missing genotype can be simulated in HAPSIMU at a rate designated by users.

**Figure 2:**
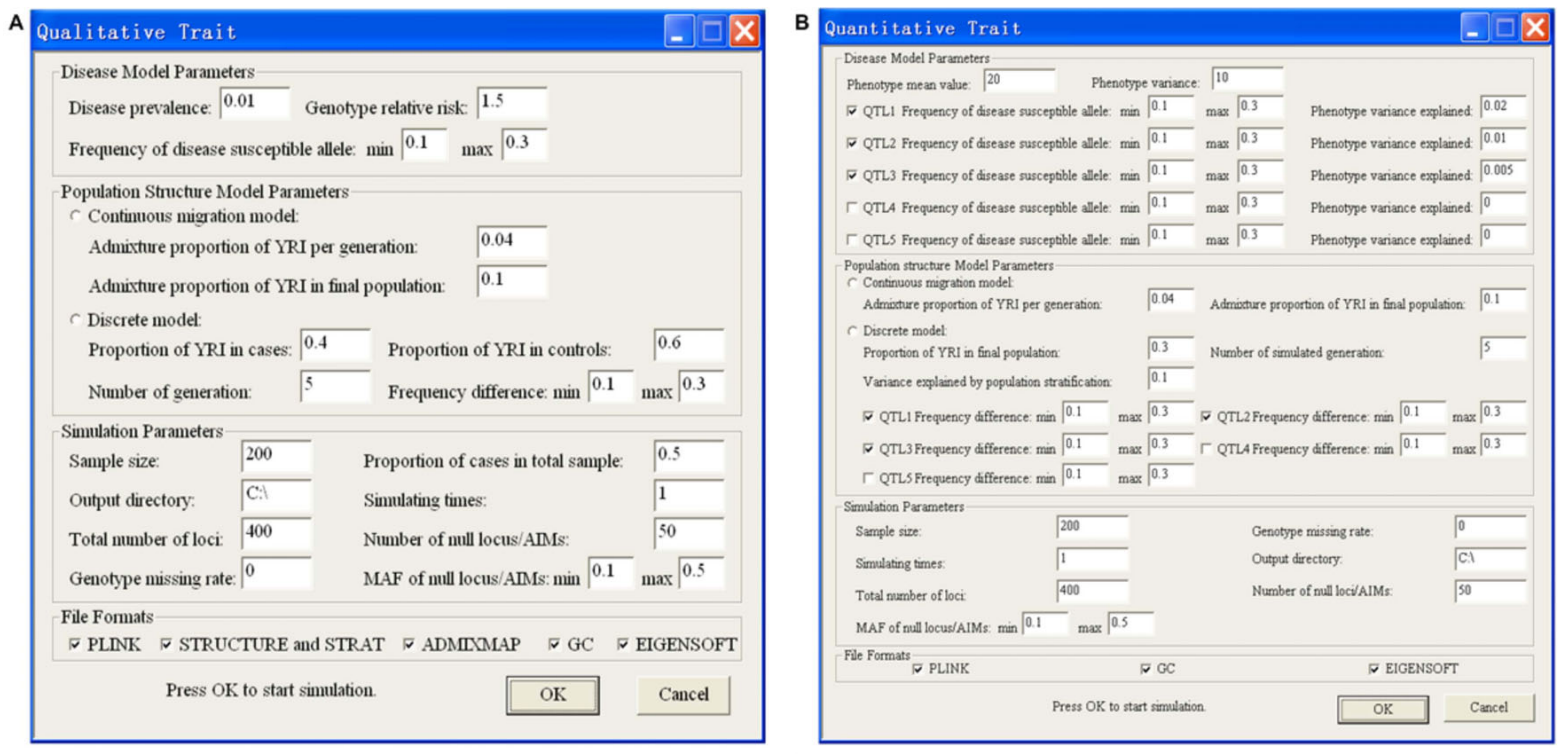
Main interface screens of HAPSIMU for qualitative (A) and quantitative (B) traits simulation.

Both qualitative and quantitative traits can be simulated in HAPSIMU using additive genetic model (Figure 2). The phenotypic effect(s) of causal locus (loci) is (are) controlled by various genetic parameters, such as number of QTLs (for quantitative trait), frequency (frequencies) of disease susceptible allele(s), disease prevalence (for qualitative trait), phenotypic variance explained by each QTL (for quantitative trait), and so on.

HAPSIMU can output the simulated data with various selectable file formats required by five prevailing PBAS software: Admixmap [15], Plink [16], STRUCTURE & STRAT [9,10], GC [7] and EIGENSOFT [8]. Currently, HAPSIMU 1.0 is designed to run on Windows operation systems. Future versions of HAPSIMU 1.0 will be able to run on Linux operation systems and to include more practical functions, for instance, future versions of HAPSIMU 1.0 can simulate heterogeneous populations using the genotype data provided by researchers in their own studies.

## Discussion

The simulated genotype and phenotype data of heterogeneous populations can be used to compare the relative performance of various PBAS methods in heterogeneous populations. The comparison results can provide a practical guideline for researchers to select proper study methods and make appropriate inference of the results in PBAS.

The simulated admixed populations can also be applied to performance comparison studies of various population structure identification and admixture mapping methods [10,15,17]. For instance, Sankararaman et al., recently developed a new method to identify population structure [17]. They simulated a set of admixed populations using the genotype data of chromosome 1 from the HapMap project, and presented the high accuracy of their new approach in population structure inference. Compared with their simulation algorithm, there are two significant differences for HAPSIMU. In Sankararaman et al.,'s study, genotype data were simulated with the same recombination fractions (10^-8^) for all base pairs, while HAPSIMU can simulate genotype data based on the real genetic map distances reported by the HapMap ENCODE project. Additionally, we selected 12,867 highly informative marker loci from 10 ENCODE regions to conduct simulations, which may further increase the effectiveness and robustness of our simulation approach for population structure.

## Conclusion

In summary, HAPSIMU provides a common genetic simulation platform for PBAS. The simulated heterogeneous populations can be used to assess the impact of population structure on PBAS, and compare the performance of various population structure identification and PBAS methods.

## Availability and requirements

**Project name**: HAPSIMU

**Project home page**: 

**Operating system**(s): Microsoft Windows

**Programming language**: C++

**License**: Free for non-commercial usage

## Authors' contributions

FZ designed and developed the HAPSIMU program. JL, JC and H–WD were responsible for the basic conception and overall project coordination. All authors have read and approved the final manuscript.

## Supplementary Material

Additional file 1 The HIPSIMU package. This zipped file contains the HAPSIMU program and user document. Click here for file

## References

[B1] Marchini J, Cardon LR, Phillips MS, Donnelly P (2004). The effects of human population structure on large genetic association studies. Nat Genet.

[B2] Risch NJ (2000). Searching for genetic determinants in the new millennium. Nature.

[B3] Lander ES, Schork NJ (1994). Genetic dissection of complex traits. Science.

[B4] Freedman ML, Reich D, Penney KL, McDonald GJ, Mignault AA, Patterson N, Gabriel SB, Topol EJ, Smoller JW, Pato CN, Pato MT, Petryshen TL, Kolonel LN, Lander ES, Sklar P, Henderson B, Hirschhorn JN, Altshuler D (2004). Assessing the impact of population stratification on genetic association studies. Nat Genet.

[B5] Guthery SL, Salisbury BA, Pungliya MS, Stephens JC, Bamshad M (2007). The structure of common genetic variation in United States populations. Am J Hum Genet.

[B6] Deng HW (2001). Population admixture may appear to mask, change or reverse genetic effects of genes underlying complex traits. Genetics.

[B7] Devlin B, Roeder K (1999). Genomic control for association studies. Biometrics.

[B8] Price AL, Patterson NJ, Plenge RM, Weinblatt ME, Shadick NA, Reich D (2006). Principal components analysis corrects for stratification in genome-wide association studies. Nat Genet.

[B9] Pritchard JK, Stephens M, Rosenberg NA, Donnelly P (2000). Association mapping in structured populations. Am J Hum Genet.

[B10] Pritchard JK, Stephens M, Donnelly P (2000). Inference of population structure using multilocus genotype data. Genetics.

[B11] Dudek SM, Motsinger AA, Velez DR, Williams SM, Ritchie MD (2006). Data simulation software for whole-genome association and other studies in human genetics. Pac Symp Biocomput.

[B12] Li C, Li M (2008). GWAsimulator: a rapid whole-genome simulation program. Bioinformatics.

[B13] Kosambi DD (1944). The estimation of map distances from recombination values. Annals of Eugenics.

[B14] Long JC (1991). The genetic structure of admixed populations. Genetics.

[B15] McKeigue PM, Carpenter JR, Parra EJ, Shriver MD (2000). Estimation of admixture and detection of linkage in admixed populations by a Bayesian approach: application to African-American populations. Ann Hum Genet.

[B16] Purcell S, Neale B, Todd-Brown K, Thomas L, Ferreira MA, Bender D, Maller J, Sklar P, de Bakker PI, Daly MJ, Sham PC (2007). PLINK: a tool set for whole-genome association and population-based linkage analyses. Am J Hum Genet.

[B17] Sankararaman S, Kimmel G, Halperin E, Jordan MI (2008). On the inference of ancestries in admixed populations. Genome Res.

