# Supplementary material for: HAPSIMU: a genetic simulation platform for population-based association studies
Source: BMC Bioinformatics. 2008 Aug 5;9:331. doi: 10.1186/1471-2105-9-331 (PMC2518933; doi:10.1186/1471-2105-9-331)
Supplement: Additional file 1 — The HIPSIMU package. This zipped file contains the HAPSIMU program and user document. [file 1471-2105-9-331-S1.zip › HAPSIMU1.0/Readme.pdf]

# Documentation for HAPSIMU 1.0

Feng Zhang[1,2], Jianfeng Liu[2], Jie Chen[3], Hong-Wen Deng[1,2,4]

1. School of Life Science and Technology, Xi'an Jiaotong University, Xi'an 710049, P R China
2. School of Medicine, University of Missouri-Kansas City, Kansas City, MO 64108, USA
3. Department of Mathematics and Statistics, University of Missouri-Kansas City, Kansas City, MO, 64110, USA
4. College of Life Sciences, Hunan Normal University, Changsha, Hunan 410081, P R China

# Contents

**1 Introduction** …………………………………………… 3

**2 Installing and Running HAPSIMU 1.0** …………. 3

**3 Simulation Method**……………………………..... 4

**4 Program Parameters** ……………………………….. 5

4.1 Qualitative trait …………………………………….. 5

4.2 Quantitative trait …………………………………… 8

**5 Output Files** …………………………………………… 10

**6 References** ……………………………………………... 13

# 1. Introduction

HAPSIMU 1.0 is a free genetic simulation platform based on real haplotype data from the HapMap ENCODE project. Under the continuous migration model or the discrete model, HAPSIMU 1.0 can simulate heterogeneous populations with various known population structures. Furthermore, both qualitative and quantitative traits can be simulated in HAPSIMU 1.0 using additive genetic model with various parameters designated by users.

HAPSIMU 1.0 can be applied as a common genetic simulation platform for population-based association studies. The simulated genotype and phenotype data of heterogeneous populations can be used to evaluate the impact of population structures on population-based association studies, and compare the relative performance of various population-based association studies methods in heterogeneous populations, which can provide a practical guideline for researchers to select optimal study approaches and make proper interpretation of their results.

# 2. Installing and Running HAPSIMU 1.0

Currently, HAPSIMU 1.0 runs on Windows operation systems. The downloaded software package includes the executable program and HapMap ENCODE haplotype data files used for simulation. Unzip the downloaded HAPSIMU 1.0 package and double clicking on the HAPSIMU 1.0 icon to start simulations.

# 3. Simulation Method

Based on the phased CEPH and YRI haplotype data and derived recombination fractions, heterogeneous populations composed of CEPH and YRI can be simulated under two different population admixture models: the continuous migration model or the discrete model. The detailed simulation procedure is illustrated in following:

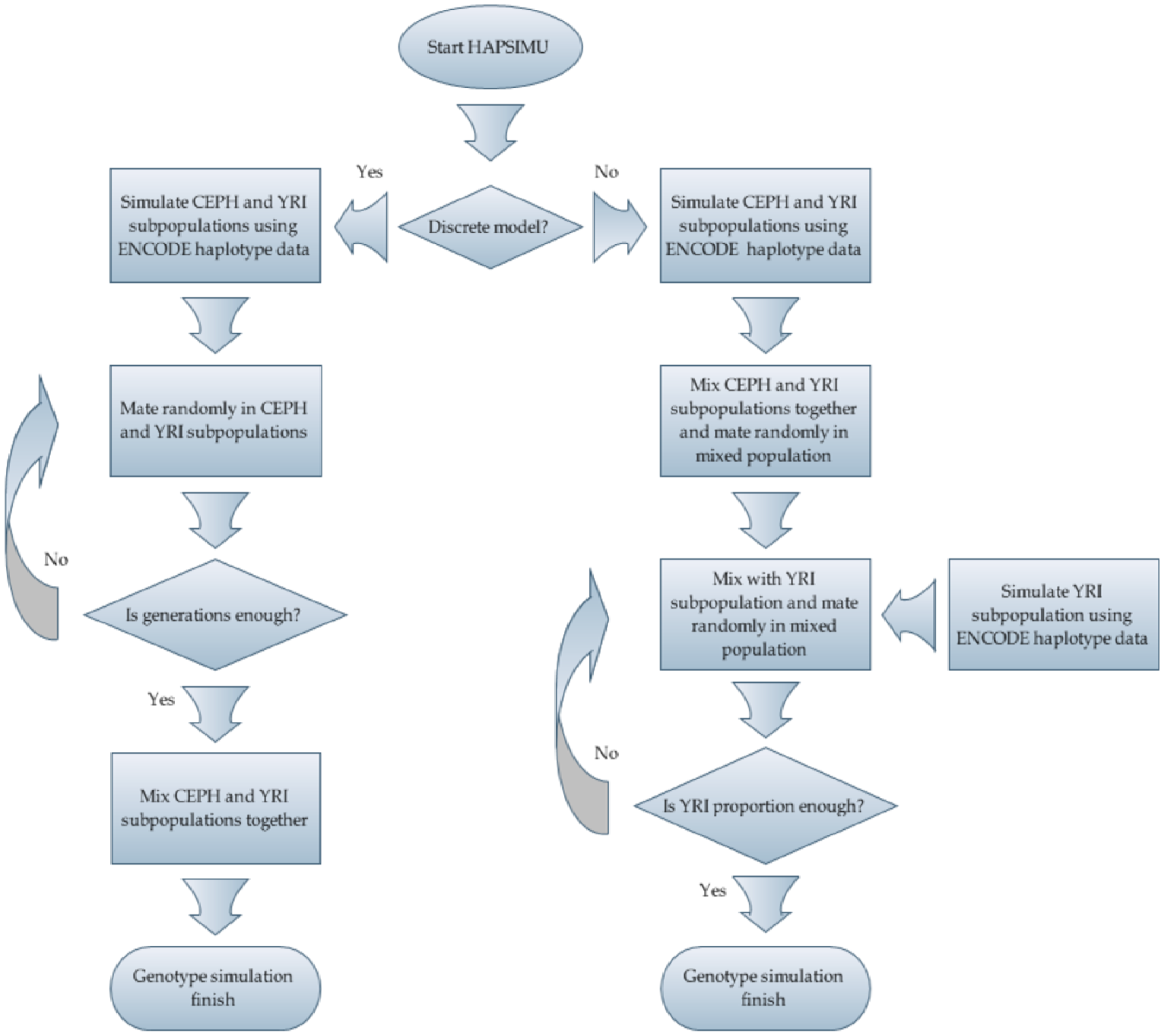

# 4. Program parameters

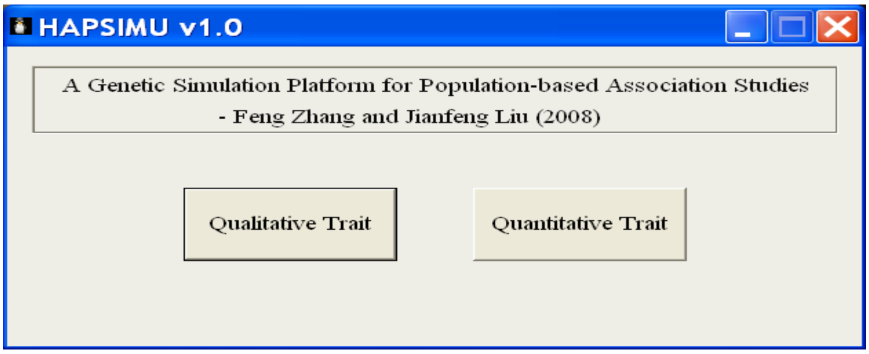


Both qualitative and quantitative traits can be simulated in HAPSIMU 1.0

## 4.1 Qualitative trait

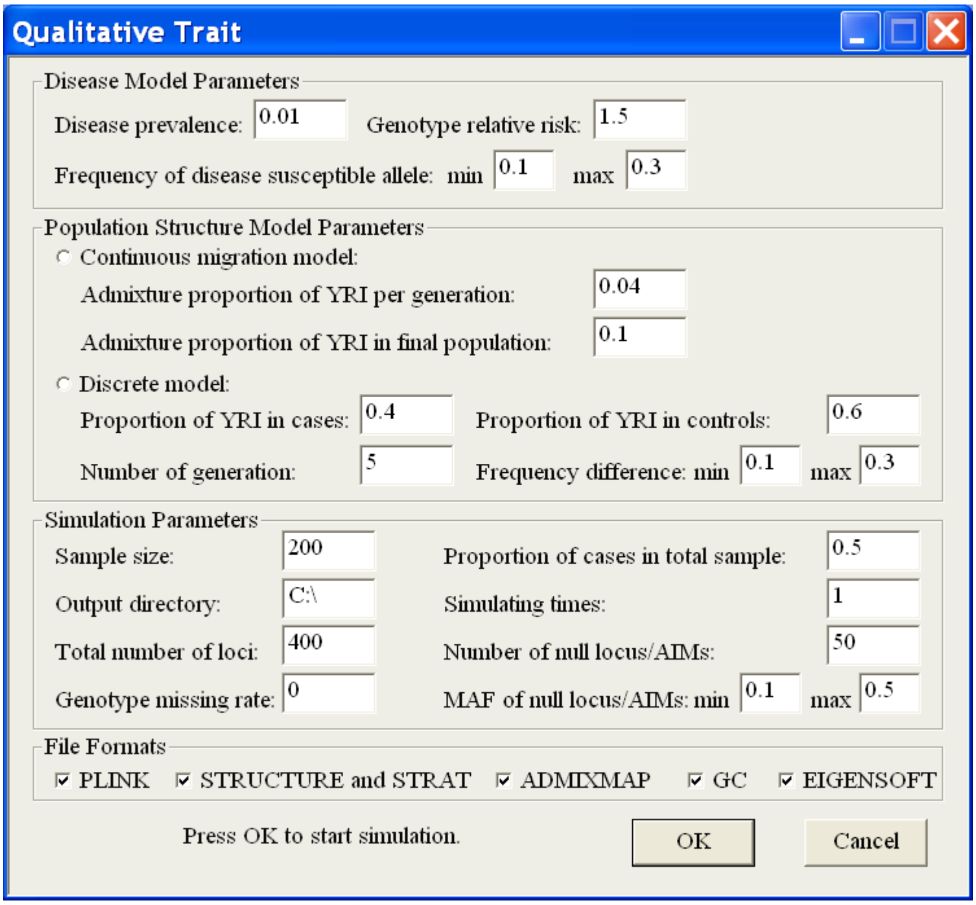

## *Disease model parameters:*

1 **Disease prevalence**: (Double) Disease prevalence in simulated heterogeneous population.

2 **Genotype relative risk**: (Double) Genotype relative risk at simulated causal locus in additive genetic model.

3 **Frequency of disease susceptible allele**: (Double) Frequency range of disease susceptible allele at simulated causal locus.

## *Population structure model parameters:*

1 **Continuous migration model**: (Boolean) Simulate heterogeneous populations under continuous migration model.

2 **Mixed proportion of YRI per generation**: (Double) Proportion of YRI individuals admixed with simulated heterogeneous populations in each generation under continuous migration model.

3 **Proportion of YRI in final population**: (Double) Final proportion of YRI individuals in simulated heterogeneous populations under continuous migration model.

4 **Discrete model**: (Boolean) Simulate heterogeneous populations under discrete model.

5 **Proportion of YRI in cases**: (Double) Proportion of YRI

individuals in case group under discrete model.

6 **Proportion of YRI in controls**: (Double) Proportion of YRI individuals in control group under discrete model.

7 **Number of generation**: (Int) Number of generation need to simulate under discrete model.

8 **Frequency difference**: (Double) Absolute value range of frequency difference of disease susceptible allele at causal locus between simulated CEPH and YRI subpopulations under discrete model.

## *Simulation parameters:*

1 **Sample size:** (Int) Number of subjects in simulated heterogeneous population.

2 **Proportion of cases in total sample**: (Double) Proportion of cases in simulated case-control group.

3 **Output directory**: (String) Output directory of simulated data.

4 **Simulating times:** (Int) Number of simulations need to conduct.

5 **Total number of loci**: (Int) Total number of loci need to simulate.

6 **Number of null loci/AIMs:** (Int) Number of null loci (for GC)/AIMs (for STRUCTURE) selected from the total loci.

7 **MAF of null loci/AIMs:** Minor allele frequency range of null

loci/AIMs.

8 **Genotype missing rate**: (Double) Genotype missing rates in simulated genotype data.

## 4.2 Quantitative trait

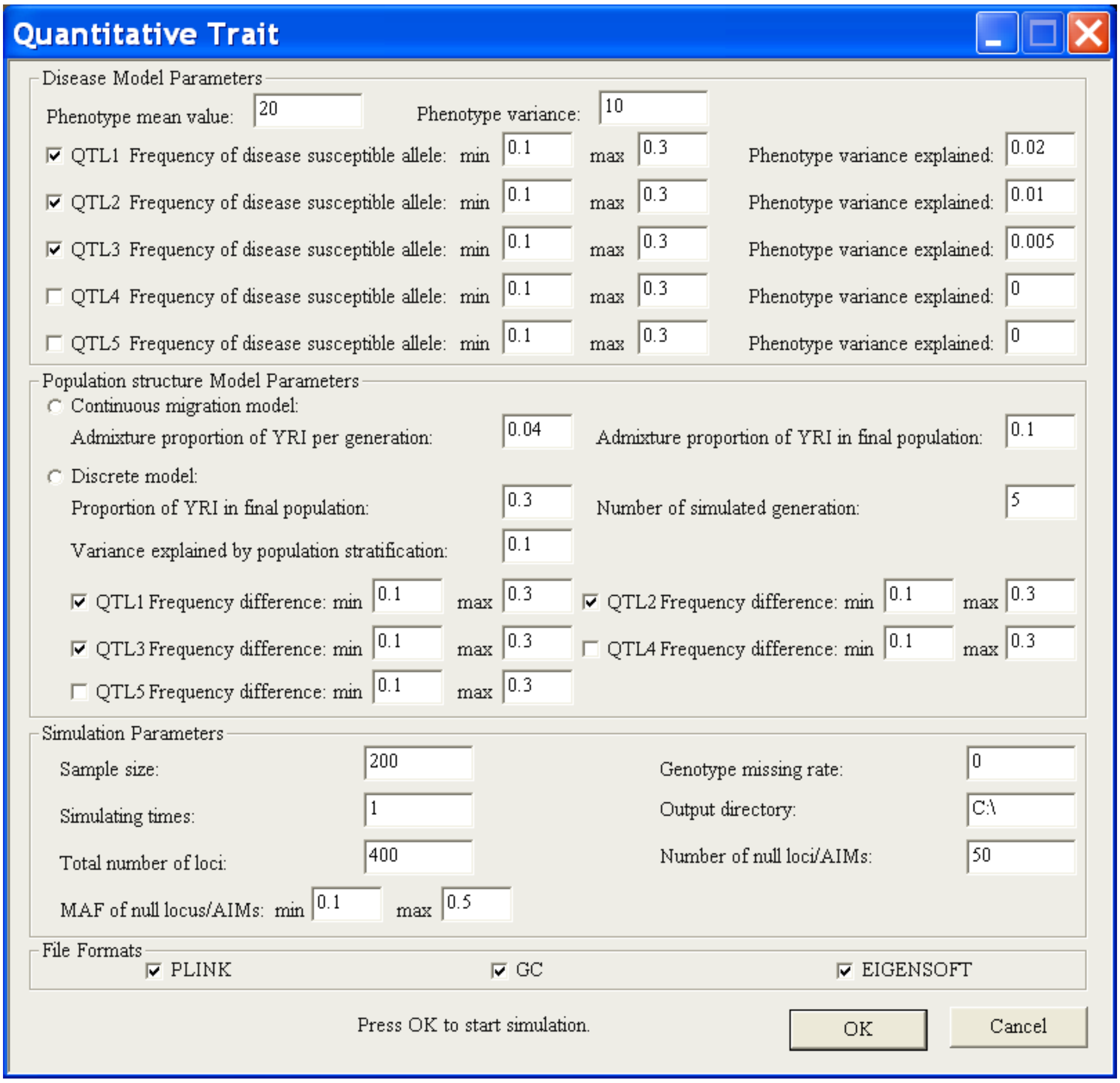


### *Disease model parameters*

1 **Phenotype mean value:** (Double) Mean value of simulated

quantitative phenotype in additive genetic model.

2 **Phenotype variance**: (Double) Variance of simulated quantitative phenotype in additive genetic model.

3 **QTL i**: (Boolean) QTL i will be simulated with preset frequency range of disease susceptible allele and explained phenotypic variance.

4 **Frequency of disease susceptible allele**: (Double) Frequency range of disease susceptible allele at QTL i.

5 **Variance explained**: (Double) Proportion of phenotypic variance explained by QTL i.

### *Population structure model parameters:*

1 **Continuous migration model**: (Boolean) Simulate heterogeneous populations under continuous migration model.

2 **Mix proportion of YRI per generation**: (Double) Proportion of YRI individuals admixed with simulated heterogeneous populations in each generation under continuous migration model.

3 **Proportion of YRI in final population**: (Double) Final proportion of YRI individuals in simulated heterogeneous populations under continuous migration model.

4 **Discrete model**: (Boolean) Simulate heterogeneous populations under discrete model.

5 **Proportion of YRI in cases**: (Double) Proportion YRI individuals in case group under discrete model.

6 **Proportion of YRI in controls**: (Double) Proportion of YRI individuals in control group under discrete model.

7 **Number of generation**: (Int) Number of generation need to simulate under discrete model.

8 **Variance explained by population stratification**: (Double) Phenotypic variance explained by population stratification.

9 **QTL i**: (Boolean) QTL i will be simulated with preset frequency difference.

10 **Frequency difference**: (Double) Absolute value range of frequency difference of disease susceptible allele at QTL i between simulated CEPH and YRI subpopulations under discrete model.

## *Simulation parameters*

Same as qualitative trait

# 5. Output files

HAPSIMU 1.0 can output the simulated genotype and phenotype data with five selectable file formats, required by Plink (Purcell, et al., 2007), Structure & Strat (Pritchard, et al., 2000; Pritchard, et al., 2000), GC (Devlin and Roeder, 1999), Eigensoft (Price, et al., 2006) and Admixmap (McKeigue, et al., 2000). The output files for each software are listed in the following. Detailed description of file formats can be found in the documents of corresponding software.

[1] **Plink** (http://pngu.mgh.harvard.edu/~purcell/plink/)

1. Plink_loci.txt: MAP file
2. Plink_geno.txt: PED file

[2] **Structure & Strat** (http://pritch.bsd.uchicago.edu/ software.html)

1. Structure.txt: genotype file for Structure
2. Strat.txt: genotype and phenotype file for Strat

Note: Simulated data is output with one-row format (see the document of Structure & Strat)

[3]**Eigensoft** (http://genepath.med.harvard.edu/~reich/Software.htm)

1. Eigensoft_Pca.snp: map file for program "smartpca" in Eigensoft

2. Eigensoft_Pca.geno: genotype file for program “smartpca” in Eigensoft
3. Eigensoft_Pca.ind: individual file for program “smartpca” in Eigensoft
4. Eigensoft_Strat.geno: genotype file of simulated causal locus for program “Eigenstrat” (qualitative trait analysis) or “EigenstratQTL” (quantitative trait analysis) in Eigensoft
5. Eigensoft.phen: phenotype file for program “Eigenstrat” or “EigenstratQTL” in Eigensoft

Note: We find that “Eigenstrat” or “EigenstratQTL”, which run on Linux operation system, may have trouble to analyze the data files generated under Window operation system. This is because of different end-of-file character used by Window and Linux operation systems. If “Eigenstrat” or “EigenstratQTL” cannot analyze the simulated data files, we suggest using the command “dos2unix” of Linux to convert the file format.

[4] **Admixmap** (http://homepages.ed.ac.uk/pmckeigu/admixmap/index.html)

1. Admixmap_loci.txt: Map file
2. Admixmap_geno.txt: genotype file

3. Admixmap_phen.txt: phenotype file

[5] **GC** (http://wpicr.wpic.pitt.edu/WPICCompGen/genomic _control /genomic_control.htm)

GC.txt: genotype and phenotype file for GC

Note: In the GC.txt, un-null loci are first output followed by null loci for each individual. Additionally, we find that GC, implemented with R language, may fails to analyze the markers with too small MAF. This may attributed to the inner limitation of GC or R. We suggest selecting the null loci with large MAF (For example, MAF > 0.1) and conduct GC analysis only to the loci with large MAF (See the user document of GC for detail).

In each simulation, the above generated files will be output to a unique folder, named by the number of present simulation.

Additionally, the SNP id of simulated causal loci in genotype files are recorded and output to a causal loci files, named "Causal_Loci_Plink&Admixmap&Eigensoft.txt" (for Plink, Admixmap and Eigensoft) and "Causal_Loci_Strat&GC.txt" (for Structure & Strat and GC) in each simulation. The origins of each individual in simulated heterogeneous populations are also recorded to a file, named "Origin.txt".

# 6. References


1. Devlin, B. and Roeder, K. (1999) **Genomic control for association studies**, *Biometrics*, 55, 997-1004.
2. McKeigue, P.M., Carpenter, J.R., Parra, E.J. and Shriver, M.D. (2000) **Estimation of admixture and detection of linkage in admixed populations by a Bayesian approach: application to African-American populations**, *Ann Hum Genet*, 64, 171-186.
3. Price, A.L., Patterson, N.J., Plenge, R.M., Weinblatt, M.E., Shadick, N.A. and Reich, D. (2006) **Principal components analysis corrects for stratification in genome-wide association studies**, *Nat Genet*, 38, 904-909.
4. Pritchard, J.K., Stephens, M. and Donnelly, P. (2000) **Inference of population structure using multilocus genotype data**, *Genetics*, 155, 945-959.
5. Pritchard, J.K., Stephens, M., Rosenberg, N.A. and Donnelly, P. (2000) **Association mapping in structured populations**, *Am J Hum Genet*, 67, 170-181.
6. Purcell, S., Neale, B., Todd-Brown, K., Thomas, L., Ferreira, M.A., Bender, D., Maller, J., Sklar, P., de Bakker, P.I., Daly, M.J. and Sham, P.C. (2007) **PLINK: a tool set for whole-genome association and population-based linkage analyses**, *Am J Hum Genet*, 81, 559-575.
